# Temporal and spatial analysis of visceral leishmaniasis in Brazil, by age group and sex: an ecological study, 2013–2022

**DOI:** 10.1590/1984-0462/2025/43/2025108

**Published:** 2025-12-15

**Authors:** Beatryz Schmidt Konczycki, Gustavo Cezar Wagner Leandro, Luciana Aparecida Fabriz, Luana Ficanha Pérez Alves, Rosane Meire Munhak Silva, Helder Ferreira, Adriana Zilly, Neide Martins Moreira

**Affiliations:** a Universidade Estadual do Oeste do Paraná, Foz do Iguaçu, PR, Brazil.; b Universidade Estadual de Maringá, Maringá, PR, Brazil.

**Keywords:** Visceral leishmaniasis, Incidence, Spatial analysis, Brazil, Time series studies, Leishmaniose visceral, Incidência, Análise espacial, Brasil, Estudos de séries temporais

## Abstract

**Objective:**

The aim of the study was to analyze the temporal trend and spatial distribution of the incidence rate of visceral leishmaniasis by age and sex across Brazilian states, macro-regions, and nationally, from 2013 to 2022.

**Methods:**

Ecological study with temporal and spatial analyses of cases recorded in Brazil’s national disease notification system, using Prais-Winsten regression and global and local Moran indices.

**Results:**

A total of 31,453 visceral leishmaniasis cases were recorded in Brazil in the period (mean incidence of 1.68 per 100,000). A statistically significant decreasing trend was observed in Brazil (annual percent change [APC]: -3.25, 95% confidence interval [95%CI] -5.65; -0.79), particularly among women (APC -4.12, 95%CI -6.68; -1.49) and children and adolescents (APC -5.17; 95%CI -8.59; -1.62). An increase was observed among women in Santa Catarina (APC 9.03; 95%CI 6.49; 11.64), in adults in Rio Grande do Sul (APC 7.39; 95%CI 3.47; 11.45) and Santa Catarina (APC 6.48; 95%CI 1.89; 11.27) and in older adults in Paraíba (APC 2.13; 95%CI 0.46; 3.84). Spatial analysis revealed high-risk municipality clusters in the Northeast, North, Midwest, and Southeast, with the highest incidence rate in children and adolescents.

**Conclusions:**

demographic factors such as age range (especially children and adolescents) and sex were associated with a high risk of visceral leishmaniasis, indicating a need for targeted control measures in Brazil.

## RESUMO

**Objetivo:**

Analisar a tendência temporal e a distribuição espacial da taxa de incidência de leishmaniose visceral (LV) por idade e sexo nos estados, macrorregiões e no Brasil, no período de 2013 a 2022.

**Métodos:**

Estudo ecológico com análises temporais e espaciais dos casos registrados no Sistema de Informação de Agravos de Notificação. Utilizou-se a regressão de Prais-Winsten e o índice de Moran global e local.

**Resultados:**

Foram registrados 31.453 casos de leishmaniose visceral no Brasil no período (incidência média de 1,68 por 100 mil habitantes). Foi observada tendência de redução estatisticamente significativa no Brasil (*annual percent change* — APC: -3,25; intervalo de confiança de 95% — IC95% -5,65; -0,79), especialmente entre mulheres (APC -4,12; IC95% -6,68; -1,49) e crianças e adolescentes (APC -5,17; IC95% -8,59; -1,62). Observou-se aumento entre mulheres em Santa Catarina (APC 9,03; IC95% 6,49; 11,64); em adultos no Rio Grande do Sul (APC 7,39; IC95% 3,47; 11,45) e em Santa Catarina (APC 6,48; IC95% 1,89; 11,27); e em idosos na Paraíba (APC 2,13; IC95% 0,46; 3,84). A análise espacial revelou aglomerados de municípios de alto risco nas Regiões Nordeste, Norte, Centro-Oeste e Sudeste, com a maior taxa de incidência em crianças e adolescentes.

**Conclusões:**

Fatores demográficos como faixa etária (especialmente crianças, adolescentes e jovens) e sexo foram associados a altos riscos de LV, indicando a necessidade de ações de controle direcionadas no Brasil.

## INTRODUCTION

Visceral leishmaniasis (VL) is a neglected, chronic disease that continues to be a global public health problem affecting all ages and genders, with the most significant impact among children under 10 years of age and adults over 50, as a result of immunological frailty.^1,2^ Vulnerability is increased by factors such as precarious housing, inadequate sanitation, malnutrition, and difficulty accessing health care.^3^ Transmission occurs primarily by bites from infected female phlebotomine flies, of which *Lutzomyia longipalpis* is the main vector of *Leishmania chagasi* in Brazil.^4^

Globally, from 50,000 to 90,000 new cases of VL are recorded annually. If untreated, these can progress to death in more than 95% of cases.^5^ VL is present in at least 13 countries of the Americas and is considered an endemic disease. In Latin America, 96% of cases occur in Brazil,^1^ where an average of approximately 3500 cases are recorded per year, as reported between 2017 and 2022, resulting in an incidence rate of 2.0 cases per 100,000 inhabitants, but with significant regional variations.^4^ Mean incidence remains stable, but lethality continues high, despite having slightly decreased from 9.8% in 2022 to 8.0% in 2023, remaining above the established target of 3.5%.^6^

In response to the above, the Plan of Action to Strengthen the Surveillance and Control of Leishmaniasis in the Americas 2017–2022 was launched in 2017, bringing together guidelines for surveillance, care, and control of the disease. Prominent among the goals was to reduce VL incidence by 50% by 2022, especially in areas where transmission was intense, expanding, or under control.^7^ Despite the resulting efforts, several regions of Brazil still return high rates of incidence.^3,4,8^

In that context, several researchers have investigated the spatial and temporal distribution of VL in Brazil, intending to understand the dynamics of the disease, identifying case clusters and analysing its geographical expansion over recent decades.^3,8,9,10^ Nonetheless, there remains limited knowledge about the temporal and spatial distribution of VL by age and sex across Brazil’s subdivisions, including which age groups and sexes have shown increases or decreases in incidence, as well as the locations with the highest risk of infection. This study aimed to analyze the temporal trend and spatial distribution of the incidence rate of VL by age and sex across Brazilian states, macro-regions, and nationally, from 2013 to 2022.

## METHOD

An ecological study with temporal and spatial analysis of VL incidence rates in Brazil. The units of temporal analysis were the 27 states and five macro-regions, while the units of spatial analysis were the 5570 municipalities. Brazil is the largest country in South America, with a population of approximately 203 million in 2022.^11,12^

All confirmed cases of VL recorded in Brazil’s notifiable disease information system (*Sistema de Informação de Agravos de Notificação*, Sinan)^13^ between 2013 and 2022, by the patient’s municipality of residence, were included. Cases were categorized by sex: female and male, and age group: children and adolescents (<19 years), adults (20-59 years), and older adults (>60 years),^14^ in Brazil’s 5570 municipalities. The data used in this study were collected in June 2024.

VL incidence rates for the years from 2013 to 2022 were calculated at the municipal, state, macro-regional, and national levels, based on resident population estimates by sex and age provided by the Ministry of Health.^15^ Incidence rates were calculated as the number of cases reported each year divided by the resident population during the same period and multiplied by 100,000 inhabitants.

The temporal trend analysis of the incidence of VL was conducted using the Prais-Winsten regression model. The method adjusts for first-order temporal autocorrelation, offering robust trend estimates and ensuring consistency and comparability across subgroups.^16^ The year of notification was considered the independent variable, and the annual incidence rates in the different geographical areas (Brazil, macro-regions, and states), stratified by sex (female and male) and age group (children and adolescents, adults, and older adults), were treated as dependent variables. The annual incidence rates were subjected to a base- 10 logarithmic transformation to ensure homogeneity of the residual variance. The annual percent change (APC) and corresponding 95% confidence intervals (95%CI) were estimated, and trends were classified as increasing, decreasing, or stable based on the p-value and the confidence interval limits. All analyses were performed using RStudio® software (version 4.1.2), with a significance level of a=0.05.

For spatial analysis of VL, the incidence rate was estimated based on the total number of reported cases during the study period, divided by the average annual population. The Local Empirical Bayes Smoother was applied to reduce random fluctuations in incidence rates. Unlike global smoothing, which uses a constant average based on the entire dataset, the local approach considers a subset of neighboring municipalities defined by a spatial weights matrix. In this study, we employed a first-order binary queen contiguity matrix based on municipal centroids, wherein neighboring municipalities are defined by shared borders.

Subsequently, the univariate Global Moran’s I test was applied to assess the spatial dependence of incidence rates and provide an overview of spatial autocorrelation. Then, the univariate Getis-Ord* test was used to identify local clusters, classifying them as High-High (H-H), indicating neighboring areas with high incidence or Low-Low (L-L), representing clusters of low-incidence regions.^17^ All spatial analyses were performed using GeoDA software (version 1.22.0.4), using a first-order queen-type contiguity matrix and a 5% level of significance.

Choropleth maps were plotted based on the official cartography of Brazilian municipalities, using the SIRGAS2000 reference system provided by the national bureau of statistics, the Instituto Brasileiro de Geografia e Estatística, and processed with QGIS software, version 2.18.2. VL incidence rates were categorised into0; >0–1; >1–50; >50–100; >100–500 and >500 cases per 100,000 inhabitants per year.

Ethical approval was not required for this study, as it utilized non-identifiable secondary data, in accordance with Resolutions No. 466/2012 and 510/2016 of the Brazilian National Health Council.

## RESULTS

Between 2013 and 2022, a total of 31,453 cases of VL were confirmed in Brazil, with a mean incidence rate of 1.68 per 100,000 inhabitants. During the period, the incidence rate of VL per 100,000 inhabitants showed a decreasing trend in Brazil (APC -3.25; 95%CI -5.65; -0.79; p=0.033). A decreasing trend was also observed in the Northeast and Midwest macro-regions, as well as in the states of Tocantins, Bahia, Ceará, Maranhão, Piauí, Mato Grosso, and São Paulo. The other macro-regions and states showed a stationary trend (Table 1).

**Table 1.** Temporal trend of visceral leishmaniasis incidence rate (per 100,000 inhabitants) per macro-regions and states of Brazil, 2013–2022.

Macro-regions/States	Incidence rate										APC (95%CI)	p-value	Trend
	2013	2014	2015	2016	2017	2018	2019	2020	2021	2022			
North	3.36	2.54	2.88	3.52	4.78	4.63	2.97	1.97	1.70	1.54	-3.53 (-7.77; 0.90)	0.156	S
AC	—	—	—	—	—	—	—	—	—	—	—	—	—
AP	0.14	0.00	0.00	0.13	0.00	0.12	0.00	0.12	0.00	0.23	3.34 (-1.62; 8.56)	0.226	S
AM	0.00	0.03	0.00	0.02	0.00	0.05	0.05	0.02	0.00	0.00	0.33 (-6.16; 7.27)	0.926	S
PA	3.35	2.97	3.48	4.36	6.93	6.80	4.03	2.66	2.05	1.80	-2.85 (-8.42; 3.05)	0.364	S
RO	0.23	0.06	0.00	0.00	0.00	0.00	0.06	0.00	0.00	0.06	-5.30 (-16.13; 6.93)	0.405	S
RR	4.10	3.62	4.15	7.78	6.89	3.30	2.15	2.53	3.22	2.45	-2.99 (-7.25; 1.47)	0.222	S
TO	18.87	11.82	13.07	14.42	15.61	15.50	11.76	7.48	7.53	7.09	-4.19 (-6.68; -1.64)	0.013	D
Northeast	3.58	4.34	3.81	3.23	3.86	3.87	2.81	2.18	1.83	1.91	-3.60 (-5.53; -1.63)	0.008	D
AL	0.76	1.29	1.41	0.71	1.42	3.16	2.19	1.91	1.60	0.95	2.24 (-2.80; 7.54)	0.415	S
BA	2.21	3.46	2.57	1.62	2.16	2.27	1.45	1.41	1.12	0.69	-5.56 (-7.47; -3.60)	0.001	D
CE	5.47	6.99	5.73	4.05	4.31	4.24	3.54	2.47	2.32	2.87	-4.85 (-6.05; -3.63)	0.001	D
MA	10.42	8.33	8.70	10.47	11.33	10.80	6.32	5.28	4.17	4.18	-4.35 (-7.42; -1.18)	0.028	D
PB	0.97	1.52	1.18	0.83	1.24	1.20	1.17	0.64	0.71	1.11	-1.84 (-3.88; 0.25)	0.122	S
PI	6.66	8.92	7.46	5.79	7.61	5.88	4.83	3.53	2.71	3.10	-4.99 (-6.85; -3.09)	0.001	D
PE	0.76	1.83	1.87	1.25	1.95	2.11	1.89	1.18	1.04	1.34	0.21 (-3.29; 3.84)	0.911	S
RN	2.37	2.96	2.35	2.53	2.74	2.76	2.77	1.95	1.66	2.05	-1.48 (-3.09; 0.15)	0.112	S
SE	2.28	3.02	2.94	2.38	3.28	3.29	2.78	2.20	1.92	2.35	-0.85 (-2.74; 1.07)	0.408	S
Midwest	2.26	1.74	1.50	1.40	1.66	1.24	1.26	1.17	1.24	1.37	-2.26 (-3.47; -1.03)	0.007	D
DF	0.61	0.42	0.48	0.91	0.82	0.50	0.43	0.33	0.42	0.23	-3.78 (-7.55; 0.14)	0.095	S
GO	0.65	0.87	0.85	0.72	1.02	0.82	0.78	0.67	0.56	0.28	-3.42 (-7.06; 0.37)	0.114	S
MT	1.13	0.59	0.89	0.48	0.63	0.55	0.34	0.37	0.45	0.64	-3.32 (-5.55; -1.04)	0.022	D
MS	9.43	6.76	4.98	4.77	5.49	3.93	4.53	4.34	4.90	6.30	-1.87 (-4.67; 1.01)	0.238	S
Southeast	0.66	0.68	0.77	0.87	1.27	0.68	0.51	0.42	0.38	0.39	-3.18 (-6.82; 0.61)	0.138	S
ES	0.10	0.08	0.18	0.35	0.57	0.25	0.20	0.00	0.05	0.12	-3.61 (-15.47; 9.91)	0.598	S
MG	1.69	1.90	2.29	2.70	4.14	2.06	1.45	1.21	1.13	0.99	-3.09 (-7.66; 1.70)	0.239	S
RJ	0.05	0.03	0.04	0.05	0.11	0.05	0.08	0.05	0.05	0.07	2.08 (-0.70; 4.94)	0.182	S
SP	0.46	0.41	0.37	0.36	0.41	0.32	0.26	0.23	0.19	0.25	-3.75 (-4.94; -2.55)	0.001	D
South	0.01	0.02	0.02	0.05	0.06	0.05	0.06	0.05	0.03	0.03	5.21 (-1.65; 12.54)	0.178	S
PR	0.00	0.02	0.04	0.11	0.04	0.08	0.06	0.03	0.03	0.01	-0.70 (-12.03; 12.09)	0.912	S
RS	0.02	0.04	0.01	0.02	0.07	0.05	0.06	0.08	0.04	0.04	6.41 (0.52; 12.64)	0.065	S
SC	0.02	0.00	0.00	0.03	0.06	0.00	0.04	0.04	0.01	0.05	5.28 (0.59; 10.19)	0.058	S
Brazil	1.73	1.84	1.74	1.67	2.14	1.85	1.34	1.04	0.91	0.93	-3.25 (-5.65; -0.79)	0.033	D

Temporal analysis of the VL incidence rate by sex revealed a decreasing trend in both females (APC -4.12; 95%CI -6.68; -1.49; p=0.016) and males (APC -2.86; 95%CI -5.19; -0.47; p=0.047) in Brazil. This decreasing temporal pattern for both sexes was evident in the Northeast and Midwest macro-regions, as well as in the states of Tocantins, Bahia, Ceará, Maranhão, Piauí, and São Paulo. In contrast, a decreasing trend was observed exclusively among females in Paraíba, Rio Grande do Norte, and Mato Grosso. The only state showing an increasing trend was Santa Catarina, where a significant rise was identified among females (APC 9.03; 95%CI 6.49; 11.64; p=0.001) (Table 2).

**Table 2.** Temporal trend of visceral leishmaniasis incidence rate (per 100,000 inhabitants) by sex in macro-regions and states of Brazil, 2013–2022.

Macro-regions/States	Female					Male				
	Incidence rate		APC (95%CI)	p-value	Trend	Incidence rate		APC (95%CI)	p-value	Trend
	2013	2022				2013	2022			
North	2.76	1.12	-3.96 (-8.07; 0.33)	0.108	S	3.94	1.96	-3.21 (-7.55; 1.33)	0.201	S
AC	—	—	—	—	—	—	—	—	—	—
AP	—	—	—	—	—	0.27	0.46	4.14 (-2.10; 10.78)	0.234	S
AM	0.00	0.00	2.38 (-1.41; 6.33)	0.256	S	0.00	0.00	-2.11 (-10.28; 6.80)	0.644	S
PA	2.73	1.30	-3.34 (-8.07; 2.36)	0.279	S	3.92	2.30	-2.47 (-8.17; 3.58)	0.439	S
RO	0.12	0.11	-2.80 (-7.38; 2.01)	0.966	S	0.36	0.00	-11.18 (-22.01; 1.14)	0.111	S
RR	3.72	2.55	-3.26 (-7.77; 1.46)	0.283	S	4.42	2.36	-3.11 (-8.18; 2.23)	0.281	S
TO	15.61	4.88	-4.54 (-7.41; -1.59)	0.017	D	21.92	9.28	-3.89 (-6.22; -1.51)	0.013	D
Northeast	2.43	0.99	-4.92 (-7.02; -2.77)	0.002	D	4.85	2.90	-3.07 (-4.96; -1.15)	0.014	D
AL	0.48	0.86	3.84 (-0.11; 7.94)	0.093	S	1.08	1.05	0.88 (-5.19; 7.34)	0.790	S
BA	1.81	0.36	-7.49 (-9.84; -5.08)	0.001	D	2.80	1.02	-4.90 (-6.76; -3.01)	0.001	D
CE	3.10	1.28	-6.05 (-7.62; -4.46)	0.001	D	7.94	4.55	-4.37 (-5.52; -3.20)	0.001	D
MA	6.95	2.22	-5.31 (-8.91; -1.56)	0.025	D	13.78	6.21	-3.86 (-6.75; -0.88)	0.035	D
PB	0.65	0.62	-3.81 (-6.02; -1.55)	0.011	D	1.33	1.63	-0.82 (-2.99; 1.41)	0.489	S
PI	4.67	1.53	-6.94 (-8.50; -5.35)	0.001	D	8.58	4.78	-3.95 (-6.08; -1.77)	0.008	D
PE	0.54	0.85	-1.15 (-3.91; 1.68)	0.446	S	1.00	1.87	0.84 (-3.08; 4.92)	0.690	S
RN	1.47	0.82	-4.59 (-6.91; -2.21)	0.006	D	3.38	3.34	-0.74 (-2.15; 0.70)	0.342	S
SE	2.32	0.91	-3.41 (-7.16; 0.49)	0.124	S	2.28	3.90	-0.09 (-2.19; 2.05)	0.933	S
Midwest	1.55	1.01	-2.61 (-3.67; -1.52)	0.002	D	2.97	1.74	-2.20 (-3.56; -0.83)	0.014	D
DF	0.35	0.06	-4.71 (-9.46; 0.28)	0.101	S	0.90	0.40	-3.72 (-8.58; 1.40)	0.189	S
GO	0.46	0.11	-5.37 (-10.96; 0.57)	0.113	S	0.85	0.40	-2.56 (-4.99; -0.06)	0.080	S
MT	0.82	0.28	-8.48 (-11.12; -5.77)	0.001	D	1.40	1.00	-1.54 (-4.60; 1.62)	0.364	S
MS	6.46	5.24	-1.97 (-3.94; 0.04)	0.090	S	12.34	7.39	-2.17 (-5.15; 0.91)	0.203	S
Southeast	0.46	0.22	-3.98 (-7.86; 0.07)	0.090	S	0.88	0.56	-2.78 (-6.34; 0.92)	0.178	S
ES	0.11	0.05	-1.66 (-15.81; 14.87)	0.838	S	0.11	0.20	-4.14 (-16.73; 10.35)	0.572	S
MG	1.17	0.56	-3.90 (-8.80; 1.27)	0.175	S	2.24	1.43	-2.77 (-7.22; 1.90)	0.275	S
RJ	0.02	0.07	1.96 (-3.52; 7.76)	0.511	S	0.09	0.08	2.30 (-1.57; 6.31)	0.281	S
SP	0.34	0.14	-4.65 (-5.99; -3.30)	0.001	D	0.59	0.37	-3.39 (-4.57; -2.20)	0.001	D
South	0.00	0.03	5.87 (-0.04; 12.13)	0.087	S	0.02	0.03	2.31 (-4.17; 9.22)	0.513	S
PR	0.00	0.00	-0.96 (-7.20; 5.69)	0.778	S	0.00	0.02	1.93 (-9.56; 14.89)	0.762	S
RS	0.00	0.02	3.72 (-3.97; 12.04)	0.380	S	0.04	0.07	5.68 (-2.05; 14.03)	0.192	S
SC	0.00	0.11	9.03 (6.49; 11.64)	0.001	I	0.03	0.00	-0.93 (-9.55; 8.50)	0.845	S
Brazil	1.21	0.54	-4.12 (-6.68; -1.49)	0.016	D	2.27	1.33	-2.86 (-5.19; -0.47)	0.047	D

The temporal analysis of VL incidence rates by age group revealed distinct temporal patterns between 2013 and 2022. Nationally, a significant decreasing trend was identified in children and adolescents (APC -5.17; 95%CI -8.59; -1.62; p=0.022), whereas no significant changes were observed among adults and older adults. Considering the macro-regions, the Northeast showed a decreasing trend among children, adolescents, and adults, whereas in the Midwest, this pattern was restricted to children and adolescents. At the state level, a decreasing trend across all age groups (children and adolescents, adults, and older adults) was observed in Bahia, Ceará, and São Paulo. In Piauí, a decreasing trend was identified among children and adolescents, as well as adults. Tocantins, Maranhão, Rio Grande do Norte, and Mato Grosso do Sul showed a decreasing trend limited to children and adolescents. In Paraíba, a decreasing trend was observed among adults, while an increasing trend was identified among older adults, whereas an increasing trend was observed among adults in Rio Grande do Sul and Santa Catarina (Table 3).

**Table 3.** Temporal analysis of visceral leishmaniasis incidence rate per 100,000 inhabitants by age group in macro-regions and states of Brazil, 2013–2022.

Macro-regions/States	Children and adolescents					Adults					Older adults				
	Incidence rate		APC (95%CI)	p-value	Trend	Incidence rate		APC (95%CI)	p-value	Trend	Incidence rate		APC(95%CI)	p-value	Trend
	2013	2022				2013	2022				2013	2022			
North	5.60	2.02	-4.72 (-9.49; 0.29)	0.102	S	1.86	1.19	-1.84 (-5.84; 232)	0.406	S	1.87	1.87	0.44 (-4.10; 5.20)	0.857	S
AC	—	—	—	—	—	—	—	—	—	—	—	—	—	—	—
AP	0.31	0.00	-2.68 (-14.43; 10.68)	0.690	S	0.00	0.41	5.07 (-6.63; 18.23)	0.436	S	0.00	0.00	1.49 (-12.57; 1780)	0.851	S
AM	0.00	0.00	1.12 (-4.79; 740)	0.726	S	0.00	0.00	-0.63 (-10.49; 1032)	0.909	S	—	—	—	—	—
PA	5.96	2.48	-4.06 (-1031; 2.63)	0.263	S	1.56	132	-0.65 (-630; 534)	0.833	S	1.65	2.18	0.39 (-4.58; 5.62)	0.885	S
RO	0.34	0.00	-7.74 (-15.72; 1.00)	0.119	S	0.11	0.00	-7.93 (-16.75; 1.81)	0.146	S	0.77	0.54	-0.84 (-16.19; 1731)	0.924	S
RR	7.76	4.45	-2.67 (-743; 233)	0320	S	1.56	131	-2.74 (-8.43; 331)	0393	S	0.00	2.14	13.98 (-2.20; 32.83)	0.132	S
TO	31.25	9.27	-5.86 (-8.44; -3.21)	0.003	D	12.06	5.92	-2.61 (-5.21; 0.07)	0.092	S	9.07	6.75	0.96 (-4.13; 632)	0.727	S
Northeast	6.27	2.05	-5.58 (-8.43; -2.65)	0.006	D	2.28	1.91	-1.92 (-339; -0.42)	0.037	D	1.76	1.64	-1.34 (-3.20; 0.57)	0.205	S
AL	1.30	1.30	1.02 (-4.18; 6.50)	0.716	S	0.47	0.85	3.90 (-1.04; 9.08)	0.162	S	033	0.51	5.85 (-0.12; 12.18)	0.091	S
BA	4.46	1.04	-6.93 (-9.08; -4.73)	0.001	D	1.26	0.50	-4.26 (-6.46; -2.00)	0.006	D	0.94	0.73	-2.10 (-3.47; -0.71)	0.018	D
CE	7.81	2.32	-7.71 (-9.82; -5.55)	0.001	D	4.18	2.95	-3.43 (-4.40; -2.46)	0.001	D	4.56	3.69	-235 (-4.22; -0.45)	0.042	D
MA	18.19	4.53	-6.33 (-10.48; -1.98)	0.022	D	5.44	439	-1.24 (-339; 0.96)	0300	S	3.23	1.97	-2.91 (-700; 135)	0.215	S
PB	1.24	1.28	-2.30 (-631; 1.89)	0310	S	0.94	1.08	-2.34 (-4.18; -0.46)	0.041	D	0.42	0.87	2.13 (0.46; 3.84)	0.037	
PI	10.68	3.80	-6.52 (-931; -3.64)	0.002	D	4.79	3.07	-3.47 (-5.14; -1.77)	0.004	D	2.24	1.61	-3.32 (-733; 0.86)	0.156	S
PE	1.35	1.39	-1.67 (-5.24; 2.03)	0398	S	0.42	138	2.67 (-1.60; 713)	0.259	S	0.69	1.08	0.61 (-1.80; 3.09)	0.635	S
RN	3.11	1.30	-4.25 (-6.95; -1.46)	0.018	D	2.14	236	-0.69 (-2.10; 0.73)	0368	S	1.60	2.29	0.04 (-331; 3.51)	0.981	S
SE	3.81	2.14	-2.17 (-5.29; 1.04)	0.219	S	1.66	2.27	-0.69 (-3.01; 1.68)	0.581	S	0.48	330	3.57 (-0.27; 756)	0.106	S
Midwest	2.31	1.03	-434 (-5.99; -2.66)	0.001	D	2.00	1.40	-1.21 (-3.01; 0.63)	0.232	S	3.62	2.03	-2.95 (-5.91; 0.09)	0.093	S
DE	0.81	0.00	-11.15 (-22.50; 1.85)	0.128	S	0.48	0.26	-2.82 (-754; 2.14)	0.293	S	0.84	0.55	-0.57 (-13.28; 14.02)	0.937	S
GO	1.03	0.29	-5.17 (-9.62; -0.50)	0.062	S	0.45	0.24	-2.45 (-5.75; 0.97)	0.196	S	0.62	0.44	0.76 (-4.13; 5.89)	0.774	S
MT	1.01	0.82	-3.98 (-774; -0.08)	0.081	S	1.08	0.53	-2.49 (-4.61; -033)	0.054	S	1.79	0.73	0.58 (-10.57; 13.12)	0.926	S
MS	8.56	4.13	-4.62 (-6.98; -2.20)	0.006	D	8.82	6.91	-0.84 (-4.76; 3.24)	0.694	S	14.95	8.59	-3.49 (-6.53; -035)	0.062	S
Southeast	0.91	0.39	-4.84 (-9.90; 0.50)	0.113	S	0.55	037	-2.58 (-5.46; 039)	0.126	S	0.64	0.44	-2.22 (-536; 1.01)	0.213	S
ES	0.17	0.09	-1.36 (-16.80; 16.94)	0.879	S	0.09	0.13	-4.43 (-1766; 10.92)	0.568	S	0.00	0.16	-6.56 (-15.22; 2.99)	0.209	S
MG	2.17	1.03	-4.18 (-10.08; 2.10)	0.224	S	1.50	0.94	-2.86 (-6.46; 0.88)	0.170	S	1.52	1.09	-2.24 (-6.84; 2.58)	0383	S
RJ	0.00	0.11	6.27 (-0.72; 13.74)	0.118	S	0.09	0.08	2.02 (-133; 5.49)	0.274	S	0.00	0.00	0.54 (-718; 8.90)	0.898	S
SP	0.71	0.23	-7.91 (-11.61; -4.06)	0.004	D	032	0.23	-2.18 (-3.43; -0.93)	0.010	D	0.53	034	-2.30 (-3.29; -1.29)	0.002	D
South	0.01	0.03	4.17 (-5.90; 1532)	0.453	S	0.01	0.04	6.11 (0.26; 1231)	0.074	S	0.00	0.02	-3.68 (-8.91; 1.84)	0.224	S
PR	0.00	0.00	-0.77 (-10.14; 9.58)	0.883	S	0.00	0.02	2.01 (-10.07; 15.71)	0.765	S	0.00	0.00	-6.21 (-11.01; -1.15)	0.044	D
RS	0.03	0.04	3.67 (-8.62; 1762)	0.591	S	0.02	0.05	7.39 (3.47; 11.45)	0.006	1	0.00	0.05	-1.53 (-727; 4.56)	0.628	S
SC	0.00	0.05	3.49 (-5.19; 12.97)	0.465	S	0.03	0.07	6.48 (1.89; 11.27)	0.023		—	—	—	—	—
Brazil	3.02	1.06	-5.17 (-8.59; -1.62)	0.022	D	1.15	0.89	-1.62 (-3.41; 0.20)	0.119	S	1.07	0.84	-1.70 (-3.71; 036)	0.144	S

**Figure 1.**
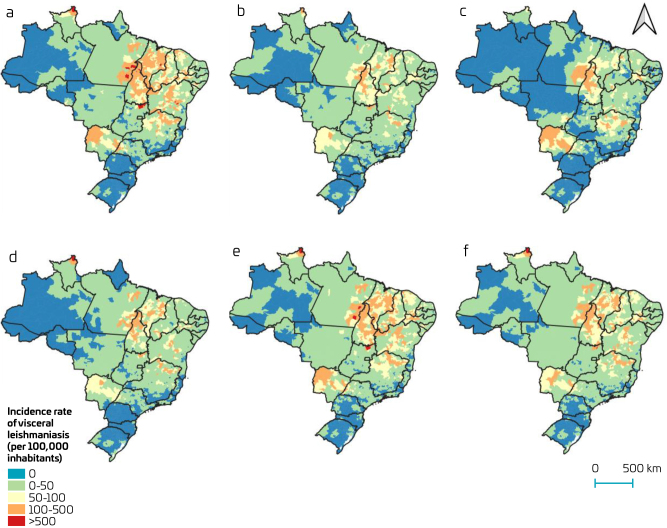
Incidence rate of visceral leishmaniasis (per 100,000 inhabitants).

Source: Figure prepared by the authors from Sinan data (2013–2022).

Spatial analysis at the municipal level revealed that 15 municipalities recorded VL incidence rates exceeding 500 cases per 100,000 children and adolescents. These included Uiramutã (Roraima); Teresina de Goiás, Monte Alegre de Goiás, and Cavalcante (Goiás); Nova Redenção and Marcionílio Souza (Bahia); Eldorado do Carajás, Redenção, São Geraldo do Araguaia, Canaã dos Carajás, and Pau D’Arco (Pará); Carmolândia and Darcinópolis (Tocantins); São João das Missões (Minas Gerais) and Buriti Bravo (Maranhão) (Figure 1A).

Among adults, no municipality reported incidence rates higher than 500 cases per 100,000 adult inhabitants (Figure 1B). In contrast, among older adults, only one municipality, Nazaré (Tocantins), exceeded this threshold (Figure 1C). With regard to sex, six municipalities recorded incidence rates above 500 cases per 100,000 male inhabitants: Uiramutã (Roraima); Teresina de Goiás and Cavalcante (Goiás), and Redenção, Pau D’Arco, and Eldorado do Carajás (Pará) (Figure 1D). Among females, only Uiramutã (Roraima) surpassed this rate (Figure 1E). Considering the overall population, VL incidence rates above 500 cases per 100,000 inhabitants were observed in two municipalities: Uiramutã (Roraima) and Teresina de Goiás (Goiás) (Figure 1F).

**Figure 2.**
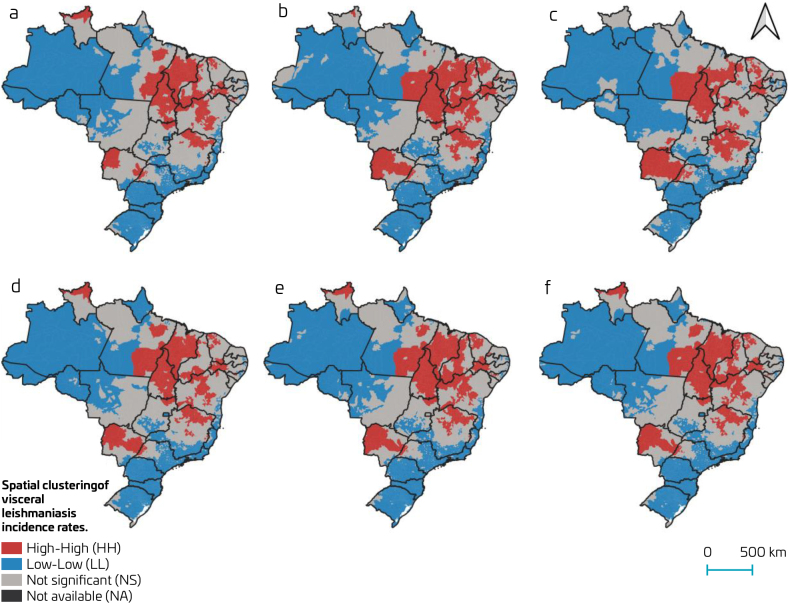
Spatial clustering of the incidence rate of visceral leishmaniasis.

Source: Figure prepared by the authors from Sinan data (2013–2022).

Spatial dependence was observed in VL incidence rates across multiple population strata, with the highest values found among adults (Moran’s I: 0.736; Figure 2B), older adults (Moran’s I: 0.730; Figure 2C), males (Moran’s I: 0.706; Figure 2D), children and adolescents (Moran’s I: 0.667; Figure 2A), total population (Moran’s I: 0.677; Figure 2F), and females (Moran’s I: 0.655; Figure 2E).

High-incidence clusters were identified mainly in the Northeast-North-Southeast regions and some isolated clusters in the North and Midwest (Figure 2F). Low incidence clusters were observed in the South, Southeast, and North regions (Figure 2F). Particularly notable were the clusters of high incidence among children and adolescents in Roraima (Figure 2A), among adults in the Northeast-North-Southeast macro-regions (Figure 2B), and among older adults in the state of Mato Grosso do Sul (Figure 2C). No substantial differences were observed between clusters when stratified by sex; however, a greater spatial concentration was noted among males (Figure 2).

## DISCUSSION

The study identified a decreasing trend in VL incidence in Brazil from 2013 to 2022, with significant variations by sex and age across different subnational divisions. Increasing incidence trends were observed in Santa Catarina (among females and adults), Rio Grande do Sul (only in adults) and Paraíba (only in older adults). Conversely, a significant decline was noted among children and adolescents nationally, particularly in the Northeast and Midwest regions. Despite this, several municipalities in Pará, Goiás, Bahia, Tocantins, Roraima, Maranhão, and Minas Gerais maintained high incidence rates for children and adolescents.

Although overall progress has been made in VL control in Brazil, the continued high burden among children and adolescents became evident after stratifying the temporal analysis by geographic location and social strata. Globally, mortality rates from VL are highest among children under 5 years old and decrease progressively with age.^18^ In the Americas, excluding Brazil, the highest incidence occurs among the under 5 years old.^6^ This increased susceptibility among children and adolescents is possibly due to inadequate health conditions and limited access to medical services.^8,19^ Our study identified a concentration of VL cases among children in the Legal Amazon, where deforestation has also been linked to increased transmission, posing additional risks to child health in the region.^20^ Given their heightened vulnerability, the clinical manifestations of VL, such as prolonged fever, weight loss, weakness, hepatospleno-megaly, anemia, and immune system dysfunction, are particularly harmful to the physical, educational, and social development of children and adolescents.^5,21,22^

The reduction in VL incidence among adults in the Northeast reflects advances in regional control strategies. However, disparities persist in Brazil, with some states having incidence rates significantly higher than the national rate, Tocantins, Ceará, Maranhão, Piauí, and Mato Grosso do Sul, and others experiencing increasing trends, including Rio Grande do Sul and Santa Catarina. These patterns highlight the need for policies that are tailored to local realities and adjusted to demographic aspects such as age. India and Sudan have implemented integrated strategies, including early treatment, vector control, and improvements in living conditions for vulnerable populations, demonstrating the effectiveness of regionally adapted approaches.^23^ The same temporal pattern was observed among older adults, with results revealing a predominantly stationary trend at national and subnational levels, indicating persistent transmission and limited progress in altering the disease’s trajectory over time. Moreover, older adults are more susceptible to complications, such as disseminated intravascular coagulation and hemophagocytic lymphohistiocytosis, which are potentially fatal conditions.^24^

The observed disparity in incidence rates, with men exhibiting higher rates and a smaller reduction over time, underscores the importance of sex as a critical factor in the disease process and highlights persistent challenges in implementing effective care and control measures at both national and state levels. The reduction was less pronounced among men, possibly due to greater exposure to the vector during outdoor activities and lower use of health care services compared to women, which may influence case severity.^25^

A reduction in incidence was observed during the pandemic period, suggesting a possible impact of COVID-19 on disease surveillance, reporting, or transmission dynamics. Similar findings were observed in Colombia and Venezuela between 2017 and 2022.^6^ The COVID-19 pandemic significantly affected VL surveillance and control, as social distancing measures disrupted key activities, including canine seroepide-miological surveys, active case detection, and medication distribution.^26,27,28^ This disruption compromised timely case detection and may have resulted in underreporting, thus masking the true incidence of the disease during the pandemic period.

The similarity between the results of this study, obtained using smoothing techniques, and those of other research based on raw incidence rates highlights the robustness of the findings.^8^ This approach is particularly important, as these clusters are located in areas with limited healthcare services, where underreporting of VL is more likely to occur.^29^ Other studies on the incidence of tegumentary leishmaniasis and the burden of cutaneous leishmaniasis have also employed this technique, revealing spatial concentrations primarily in the North and Northeast regions.^30^ The spatial cluster persistence is associated with the presence of vectors, precarious socioeconomic conditions, vulnerability in peripheral and rural areas, human mobility, and deforestation.^10,22^

Although the smoothing technique was applied and residual analysis indicated no significant spatial bias, the use of secondary data may still introduce information bias in the VL incidence rate by favoring municipalities with more coverage and sensitive epidemiological surveillance systems for case detection. Despite these challenges, Sinan remains the main national population-based surveillance system, with compulsory notification and broad adherence throughout the country, serving as a valuable tool for monitoring and analyzing the epidemiological profile of VL in Brazil. Additionally, the COVID-19 pandemic has limited the interpretation of changes in incidence rates by affecting surveillance and control activities, particularly in the final years of the series, potentially favoring the identification of decreasing trends. Future studies employing counterfactual approaches should be pursued to better inform and guide interventions tailored to different social strata. Additionally, space–time analyses using high-granularity units, such as health regions or municipalities, are needed to support more effective, context-specific local policy planning.

Finally, this study analyzed the spatial heterogeneity of VL incidence in Brazil, identifying clusters of high transmission and variations in temporal trends across social strata and geographic regions. Although VL incidence rates declined between 2013 and 2022, the disease remains a significant public health concern, particularly in municipalities with sustained high transmission. Clusters were concentrated in the Northeast, North, and Southeast regions, as well as in isolated areas of the North and Midwest. While some states demonstrated effective control across all social strata, others continue to face difficulties in reducing incidence. The identification of priority areas and vulnerable populations provides crucial guidance for strengthening control measures and enhancing public health responses to VL in Brazil. These findings underscore persistent social and territorial inequalities and reinforce the need for regionally tailored strategies.

## Data Availability

The database that originated the article is available with the corresponding author.

## References

[1] (2024). Organizaç¡o Pan-Americana da Saÿde [homepage on the Internet]. Leishmanioses: informe epidemiol—gico da Regi¡o das Am«ricas. n. 13.. https://iris.paho.org/handle/10665.2/63166.

[2] Elmahallawy EK, Alkhaldi AA, Saleh AA (2021). Host immune response against leishmaniasis and parasite persistence strategies: A review and assessment of recent research. Biomed Pharmacother.

[3] Melo SN, Barbosa DS, Bruhn FR, Câmara DC, Simões TC, Buzanovsky LP (2023). Spatio-temporal relative risks and priority areas for visceral leishmaniasis control in Brazil, between 2001 and 2020. Acta Trop.

[4] (2022). Brasil. Ministério da Saúde [homepage on the Internet].. Situação epidemiológica da Leishmaniose Visceral..

[5] World Health Organization [homepage on the Internet]. (2023). Leishmaniose.

[6] (2023). Organização Pan-Americana da Saúde [homepage on the Internet].. Leishmanioses: Informe epidemiológico das Américas. n. 12..

[7] (2017). Plan de acción para fortalecer la vigilancia y control de las leishmaniasis en las Américas 2017-2022..

[8] Nina LN, Caldas AJ, Soeiro VM, Ferreira TF, Silva TC, Rabelo PP (2023). Spatial-temporal distribution of visceral leishmaniasis in Brazil from 2007 to 2020. Rev Panam Salud Publica.

[9] Santos CJ, Santos MM, Lins FC, Silva JP, Lima KC (2023). Temporal trend in the incidence of human visceral leishmaniasis in Brazil. Cien Saude Colet.

[10] Prestes-Carneiro LE, Daniel LA, Almeida LC, D’Andrea LZ, Vieira AG, Anjolete IR (2019). Spatiotemporal analysis and environmental risk factors of visceral leishmaniasis in an urban setting in São Paulo State Brazil.. Parasit Vectors.

[11] (2017). Instituto Brasileiro de Geografia e Estatística [homepage on the Internet].. Divisão Regional do Brasil..

[12] (2022). SInstituto Brasileiro de Geografia e Estatística [homepage on the Internet]. Cidades e Estados.

[13] (2024). Brasil. Ministério da Saúde. DATASUS [homepage on the Internet].. Tabnet..

[14] (2007). Brasil. Ministério da Saúde. Secretaria de Atenção à Saúde. área de Saúde do Adolescente e do Jovem [homepage on the Internet].. Marco legal: saúde, um direito de adolescentes/Ministério da Saúde, Secretaria de Atenção à Saúde, Área de Saúde do Adolescente e do Jovem. Brasília: Editora do Ministério da Saúde.

[15] (2022). Brasil. Ministério da Saúde [homepage on the Internet].. Estimativas de população..

[16] Prais SJ, Winsten CB (1954). [homepage on the Internet].. Trend estimators and serial correlation..

[17] Duncan EW, White NM, Mengersen K. (2017). Spatial smoothing in Bayesian models: a comparison of weights matrix specifications and their impact on inference.. Int J Health Geogr.

[18] Zhang SX, Yang GB, Sun JY, Li YJ, Yang J, Wang JC (2025). Global, regional and national burden of Visceral leishmaniasis, 1990-2021: findings from the global burden of disease study 2021.. Parasit Vectors.

[19] ávila IR, Araújo GR, Barbosa DS, Bezerra JM (2023). Occurrence of human visceral leishmaniasis in the Central-West region of Brazil: a systematic review.. Acta Trop.

[20] Hage RS, Silva SV, Bohm BC, Lima JV, Bruhn NC, Menezes GR (2024). Spatiotemporal relationship between agriculture, livestock, deforestation, and visceral leishmaniasis in Brazilian legal Amazon.. Sci Rep.

[21] Resende MC, Xavier PB, Ferreira MA, Franco RT, Vilar KT, Cabral AM (2024). Leishmaniose visceral em crianças: aspectos clínicos e epidemiológicos. REAS.

[22] Liao H, Lyon CJ, Ying B, Hu T (2024). Climate change. its impact on emerging infectious diseases and new technologies to combat the challenge.. Emerg Microbes Infec.

[23] Joshi AB, Banjara MR, Chuke S, Kroeger A, Jain S, Aseffa A (2023). Assessment of the impact of implementation research on the Visceral Leishmaniasis (VL) elimination efforts in Nepal.. PLoS Negl Trop Dis.

[24] Driemeier M, Oliveira PA, Druzian AF, Brum LF, Pontes ER, Dorval ME (2015). Late diagnosis: a factor associated with death from visceral leishmaniasis in elderly patients. Pathog Glob Health.

[25] Van der Auwera G, Davidsson L, Buffet P, Ruf MT, Gramiccia M, Varani S (2022). Surveillance of leishmaniasis cases from 15 European centres. 2014 to 2019: a retrospective analysis.. Euro Surveill.

[26] Silveira JR, Lima SV, Santos AD, Siqueira LS, Santos GR, Sousa AF (2024). Impact of the COVID-19 pandemic surveillance of visceral leishmaniasis in Brazil: an ecological study.. Infect Dis Rep.

[27] Paul A, Singh S (2023). Visceral leishmaniasis in the COVID-19 pandemic era. Trans R Soc Trop Med Hyg.

[28] Uwishema O, Sapkota S, Wellington J, Onyeaka CV, Onyeaka H (2022). Leishmaniasis control in the light of the COVID-19 pandemic in Africa.. Ann Med Surg (Lond).

[29] Ribeiro CJ, Santos AD, Lima SV, Silva ER, Ribeiro BV, Duque AM (2021). Space-time risk cluster ofvisceral leishmaniasis in Brazilian endemic region with high social vulnerability: an ecological time series study. PLoS Negl Trop Dis.

[30] Melo SN, Barbosa DS, Câmara DC, Simões TC, Buzanovsky LP, Duarte AG (2024). Tegumentary leishmaniasis in Brazil: priority municipalities and spatiotemporal relative risks from 2001 to 2020. Pathog Glob Health.

